# Antineuroinflammatory Effects of Modified Wu-Zi-Yan-Zong Prescription in *β*-Amyloid-Stimulated BV2 Microglia via the NF-*κ*B and ERK/p38 MAPK Signaling Pathways

**DOI:** 10.1155/2017/8470381

**Published:** 2017-07-17

**Authors:** Qian Yu, Fang-Jiao Song, Jin-Feng Chen, Xin Dong, Yong Jiang, Ke-Wu Zeng, Peng-Fei Tu, Xue-Mei Wang

**Affiliations:** ^1^Research Studio of Integration of Traditional and Western Medicine, First Hospital, Peking University, Beijing 100034, China; ^2^State Key Laboratory of Natural and Biomimetic Drugs, School of Pharmaceutical Sciences, Peking University, Beijing 100191, China

## Abstract

Modified Wu-Zi-Yan-Zong prescription (MWP), a traditional Chinese medicinal decoction, has possessed the neuroprotective and anti-inflammatory properties. The mechanisms associated with these properties, however, are not completely understood. We designed the experiments to elucidate the antineuroinflammatory property of MWP in BV2 microglia activated by *β*-amyloid (A*β*), which is a characteristic feature of Alzheimer's disease (AD). The composition of MWP was studied using HPLC. BV2 microglia cells were then treated with A*β* in the presence or absence of MWP. The effects of MWP treatment on A*β*-activated neuroinflammation were determined using PCR, western blotting, and immunofluorescence staining. MWP significantly inhibited the mRNA expression of inflammatory mediators such as IL-1*β*, IL-6, TNF-*α*, and MCP-1, as well as the expression of inducible nitric oxide synthase (iNOS) in A*β*-activated BV2 microglia. MWP also inhibited the nuclear translocation and signaling pathway of nuclear factor kappa B (NF-*κ*B) by suppressing inhibitor of nuclear factor-*κ*B (I*κ*B) degradation and downregulating I*κ*B kinase *β* (IKK*β*) phosphorylation. Moreover, MWP decreased extracellular regulated protein kinase (ERK)/p38 mitogen-activated protein kinase (MAPK) phosphorylation, which is an important signaling pathway for proinflammatory gene expression. We concluded that MWP could suppress neuroinflammatory responses in A*β*-activated BV2 microglia via the NF-*κ*B and ERK/p38 MAPK signaling cascades and could prove an effective therapeutic agent for the prevention and treatment of neuroinflammatory diseases such as AD.

## 1. Introduction

Alzheimer's disease is a common neurodegenerative disease characterized by *β*-amyloid peptide, neurofibrillary tangles enriched with abnormally phosphorylated tau protein, and neuronal degeneration [1]. Neuroinflammation is a main factor in AD pathogenesis [2–4]. Microglia cells are a type of macrophage located in the CNS, which contribute to the inflammatory reactions occurring during AD pathogenesis by binding to soluble amyloid *β* (A*β*) oligomers and A*β* fibrils via cell-surface receptors [5]. Neuroinflammation is a manifestation of CNS damage and could activate microglia cells and astrocytes to release inflammatory factors and cytokines, invade the immune system, and activate the complement system [6]. Inflammatory factors such as TNF-*α*, IL-6, and IL-1*β* are released by activated microglia cells and astrocytes [7–9]. The stage of neuroinflammation is therefore significant in the study of AD.

*β*-Amyloid peptides originate from proteolysis of the amyloid precursor protein in amyloidogenic processing which is initiated by beta-site amyloid precursor protein-cleaving enzyme 1 (BACE-1) and successively cleaved by *γ*-secretase, a protein complex with presenilin 1 at its catalytic core [10]. In AD, A*β*-a small peptide composed by 39–43 amino acids forms long, insoluble amyloid fibrils, which accumulate in spherical microscopic deposits known as senile plaques [11]. A*β* deposition alone might be sufficient to induce an inflammatory reaction that subsequently contributes to cognitive decline and disease development [5]. A*β* is also therefore of considerable interest in the study of AD.

Several signaling pathways, including the nuclear factor kappa B (NF-*κ*B) and mitogen-activated protein kinase (MAPK) pathways, are associated with neuroinflammation [12, 13]. NF-*κ*B signaling pathway mediates the proceeding of neuroinflammation and nuclear translocation to release the production of inflammatory mediators [14]. The MAPK signaling pathway induces strong expression of neuronal chemokines and presents potential therapeutic targets for the treatment of inflammation-mediated CNS disorders [15].

Modified Wu-Zi-Yan-Zong prescription (MWP) is a decoction which is originated from Wu-Zi-Yan-Zong pill in Tang Dynasty and prepared following the principles of traditional Chinese medicine. It consists of 6 Chinese herbs:* Lycium barbarum* L.,* Cuscuta chinensis* Lam.,* Rubus chingii* Hu.,* Schisandra chinensis *(Turcz.) Baill.,* Plantago asiatica* L., and* Epimedium brevicornum *Maxim. The composition of MWP is shown in Table 1. The application of MWP has manifested the remarkable and effective impact in the regulation of the occurrence of AD in different mechanisms: it inhibits the phosphorylation of tau protein under the stimulation of A*β*, alleviates the neurofibrillary tangles in the brain, and reduces functional neuron damage to relieve dementia [16]. Moreover, MWP could suppress the inflammatory response in LPS-stimulated BV2 microglia to decrease the production of inflammatory factors [17]. Parts of the flavonoids and polysaccharides in MWP could penetrate the blood-brain barrier and prevent nerve injury following A*β* stimulation [18]. In addition, it has been proved that MWP and its flavonoid and polysaccharide components showed the protective function to the recession of study and memory ability of AD mice due to the damage of central cholinergic system and the deposition of A*β* [19–21]. Last but not least, the compounds in MWP also showed the powerful antineuroinflammatory effect in LPS-activated BV2 cells [17]. Although MWP has neuroprotective properties, the mechanisms underlying its antineuroinflammatory activity in A*β*-activated BV2 microglia remain unclear. The aim of this study was to observe the effect of MWP to A*β*-induced BV2 microglia and elucidate the antineuroinflammatory mechanisms of this traditional Chinese prescription.

## 2. Materials and Methods

### 2.1. Materials and Reagents

All herbs were purchased from Tong Ren Tang Chinese Medicine Co., Ltd. (Beijing, China), and carefully authenticated by Dr. P. F. Tu, Pharmacognosist, according to Chinese Pharmacopoeia (The Pharmacopoeia Commission of PRC, 2010). A*β* (1–42) were obtained from GL Biochem (Shanghai), Ltd. iNOS, p-I*κ*B, I*κ*B, p-IKK*β*, IKK*β*, p-NF-*κ*B (p65), NF-*κ*B, p-ERK, ERK, p-JNK, JNK, p-p38, p38, GAPDH, *α*-tubulin, Histone 3H polyclonal antibodies, and HRP conjugated anti-rabbit IgG were obtained from Cell Signaling Technology (Boston, MA, USA). SuperSignal West Femto Stable Peroxide substrate and AlexaFluor594-labeled goat anti-rabbit IgG antibody were obtained from Thermo Scientific (San Jose, CA, USA). 4′,6-Diamidino-2-phenylindole (DAPI) was purchased from Macgene (China).

### 2.2. Preparation of MWP Extracts

MWP consists of six medicinal components (Table 1). The MWP water extract was prepared as described in a previous report [22]. The voucher specimen was deposited at the Modern Research Center for Traditional Chinese Medicine, Peking University Health Science Center, Beijing, China.

### 2.3. HPLC Analysis

**Figure 1:**
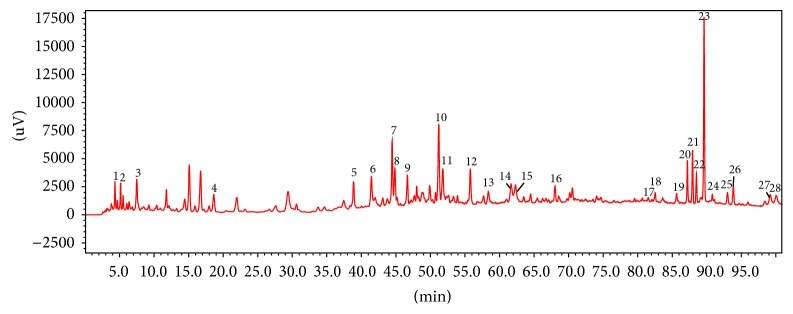
*HPLC analysis of modified Wu-Zi-Yan-Zong prescription (MWP)*. HPLC fingerprint of MWP (see Table 2).

MWP extracts were analyzed using a Shimadzu prominence liquid chromatography platform (Kyoto, Japan) equipped with two LC-20AT pumps, CTO-20A column oven, DGU-20A5R degasser, SIL-20A autosampler, and SPD20AD detector. Chromatographic separation was conducted on a AichromBond-AQ C18 column (250 mm × 4.6 mm, 5 *μ*m; Abel Industries Ltd., Canada), protected by a Phenomenex® C18 guard cartridge (3 × 4 mm, 5 *μ*m; Torrance, CA, USA). The mobile phase consisted of ACN (A) and 0.1% aqueous formic acid (B) and was delivered at 1.0 mL·min^−1^ with the following gradient program: 0–25 min, 0–3% B; 25–70 min, 3–22% B; 70–85 min, 22–35% B; 85–95 min, 35-35% B; 95–100 min, 35–100% B; 100–105 min, 100-100% B. The column was maintained at 40°C. At the end of each run, the delivery of 100% A was performed for another 14 min for system reequilibration. The monitor wavelength was set at 235 nm, 254 nm, and 280 nm, respectively.  Figure 1 and Table 2 show the HPLC fingerprint and the extracts of MWP.

### 2.4. Cell Culture

BV2 microglia were obtained from Peking Union Medical College Cell Bank (Beijing, China) and maintained in DMEM (Macgene, China) supplemented with 10% fetal bovine serum (Biowest, France) and 1% Penicillin-Streptomycin (100x, Macgene, China) in a humidified incubator containing 95% air and 5% CO_2_ at 37°C.

### 2.5. RNA Isolation and Real-Time Polymerase Chain Reaction (PCR) Analysis

BV2 microglia were treated with A*β* (20 *μ*M) in the absence or the presence of different concentrations of MWP (50, 100, and 200 mg/L) for 6 h. Total RNA was extracted using the RNAprep pure Cell Kit (Tiangen Biotech Co., Ltd, China). Reverse transcription was performed using the TIANScript RT Kit (Tiangen Biotech Co., Ltd, China) to obtain cDNA. Real-time PCR was performed in an ABI7500 real-time PCR instrument (Applied Biosystems) with the SYBR Green qPCR SuperMix, and the transcripts were amplified in a tube containing 1 *μ*g of cDNA and 0.1 *μ*mol of each forward and reverse primer. The PCR amplification procedure was as follows: 95°C for 10 min followed by 40 cycles of 95°C for 30 seconds, 54°C for 30 seconds, 72°C for 60 seconds and a final extension at 95°C for 30 seconds, 55°C for 30 seconds, and 95°C for 30 seconds. Melting curve analysis was carried out after amplification to verify the accuracy of the amplicon.

### 2.6. Western Blot Analysis

Total cell proteins were separated by SDS-PAGE and transferred onto polyvinylidene fluoride (PVDF) membranes. The PVDF membranes were then blocked with 5% nonfat milk and incubated with primary antibodies at 4°C overnight. The membranes were washed with PBST (phosphate-buffered saline, 0.1% Tween 20) for three times and incubated with secondary antibodies at room temperature for 1 h. The membranes were washed with PBST for another three times and visualized using Immobilon Western Chemiluminescent HRP Substrate. The relative optical densities were scanned using the Kodak Digital Imaging System (Gel Logic 2200Pro, Kodak, USA).

### 2.7. Immunofluorescence Analysis

BV2 microglia were seeded onto glass cover slips and treated with A*β* in the absence or the presence of MWP for 1 h. The cells were fixed with cold 4% paraformaldehyde for 20 min and permeabilized with 0.5% Triton X-100 for 30 min. The cells were then blocked with 5% BSA for 1 h and incubated with a primary antibody overnight at 4°C. The cells were washed with PBS for three times and secondary antibody labeled with Alexa Fluor-594 was added and incubated for 1 h. The cells were then washed another three times, DAPI (50 *μ*g/mL) was added for 20 min at 37°C in dark, and the coverslips were sealed. The cells were visualized using a confocal microscope (Leica TCS SP8 MP FCS, Germany).

### 2.8. Statistical Analysis

Data were represented as the mean ± SD for each group and were assessed by one-way analysis of variance (ANOVA) by using the SPSS 16.0 software (IBM Corp., USA). *P* < 0.05 compared with control group was considered statistically significant.

## 3. Results

### 3.1. Identification of the Components of MWP Extract

HPLC analysis was used to identify the ingredients of the MWP extract. Twenty-eight compounds were identified. The HPLC fingerprint of the MWP extracts and the characterization and sources of these compounds are shown and listed in Figure 1 and Table 2.

### 3.2. MWP Inhibits the mRNA Expression of Inflammatory Mediators and the Protein Expression of iNOS in A*β*-Activated BV2 Microglia

**Figure 2:**
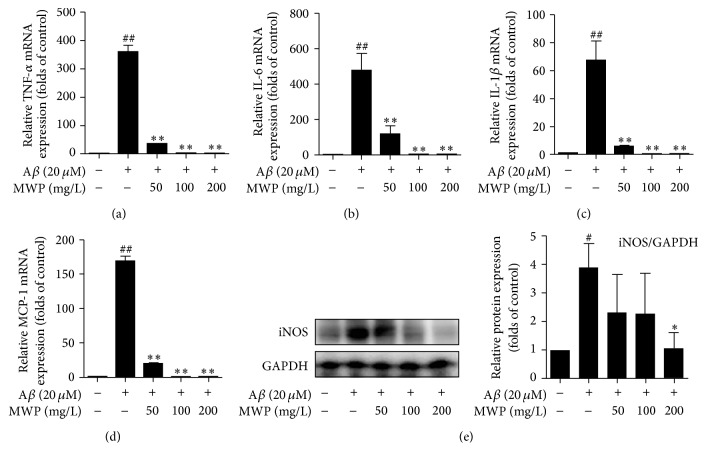
*MWP inhibits the mRNA expression of inflammatory mediators, as well as the protein expression of iNOS in Aβ-activated BV2 microglia*. BV2 microglia were incubated with A*β* (20 *μ*M) in the presence or absence of MWP (50, 100, and 200 mg/L) for 6 h. mRNA expressions of IL-6, TNF-*α*, IL-1*β*, and MCP-1 were tested (a–d). BV2 microglia were incubated with A*β* (20 *μ*M) in the presence or absence of MWP for 24 h. Total protein of the cells was extracted and used to detect the expression of iNOS by western blot (e). All values are expressed as mean ± standard deviation of triplicate tests. ^##^*P* < 0.01 and ^#^*P* < 0.05 relative to the control group; ^*∗*^*P* < 0.05 and ^*∗∗*^*P* < 0.01 relative to the A*β* group (see Table 3).

We tested the effects of MWP on the mRNA levels of IL-1*β*, IL-6, TNF-*α*, and MCP-1 by RT-PCR. The qPCR primer sequences of mRNA and the results are shown in Figures 2(a)–2(d) and Table 3. Stimulation with A*β* (20 *μ*M) for 6 h upregulated IL-1*β*, IL-6, TNF-*α*, and MCP-1 gene expression. However, MWP treatment resulted in a concentration-dependent decrease of IL-1*β*, IL-6, TNF-*α*, and MCP-1 mRNA expression in BV2 microglia. Further, as shown in Figure 2(e), treatment with A*β* (20 *μ*M) caused an increase in the protein expression of iNOS. MWP treatment significantly inhibited the increase of iNOS expression in a concentration-dependent manner. Thus, MWP showed inhibitory effects on A*β*-challenged BV2 microglia activation via the downregulation of different inflammatory mediators and inflammation-related proteins.

### 3.3. MWP Inhibits NF-*κ*B (p65) Nuclear Translocation in A*β*-Activated BV2 Microglia

**Figure 3:**
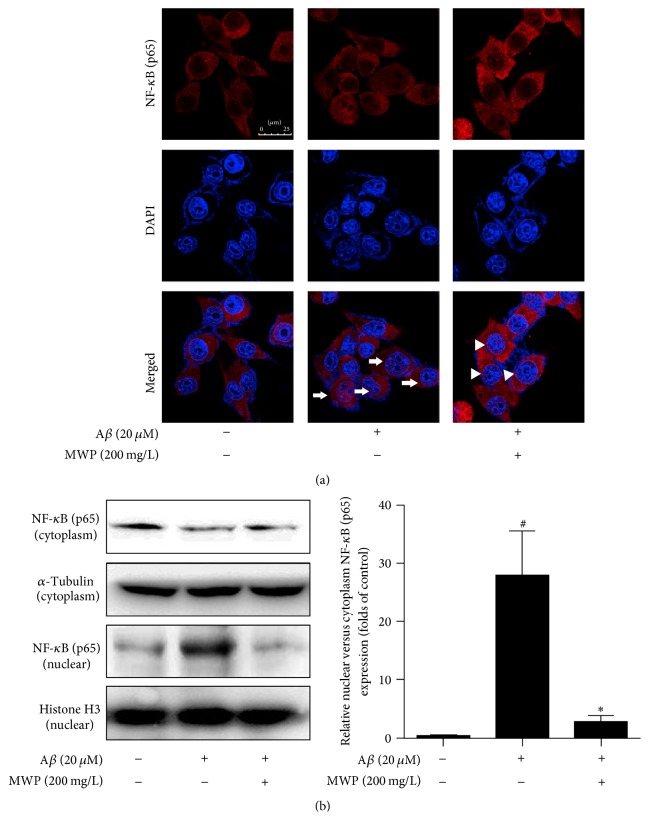
*MWP inhibits NF-κB (p65) nuclear translocation in Aβ-activated BV2 microglia*. BV2 microglia were incubated with A*β* (20 *μ*M) in the presence or absence of MWP (200 mg/L) for 1 h. Immunofluorescence analysis using an NF-*κ*B (p65) antibody was performed. We used confocal laser scanning microscopy to observe the translocation of NF-*κ*B (p65; red) into the nucleus (blue; scale bar = 25 *μ*m) (a). Western blot showed that MWP inhibited the nuclear translocation of NF-*κ*B (p65) in A*β*-activated BV-2 microglia (b). Data are presented as mean ± standard deviation from independent experiments performed in triplicate. ^#^*P* < 0.05 relative to the control group; ^*∗*^*P* < 0.05 relative to the A*β* group.

To further study the mechanism of action of MWP, we investigated the effects of MWP on crucial inflammatory signaling pathways. NF-*κ*B, a key transcription factor, was activated by A*β* and translocated to the nucleus. We first examined the nuclear translocation of NF-*κ*B in BV2 microglia by using NF-*κ*B-specific fluorescence staining and found that A*β*-stimulation induced obvious NF-*κ*B accumulation (purple fluorescence) in the nucleus. MWP treatment significantly reversed NF-*κ*B nuclear translocation; cytoplasmic retention of NF-*κ*B was more prominent in MWP-treated BV2 microglia than in A*β*-treated cells (Figure 3(a)). NF-*κ*B nuclear translocation was blocked by MWP at a dosage of 200 mg/L in BV2 microglia (Figure 3(b)).

### 3.4. MWP Inhibits NF-*κ*B Signaling by Preventing the Phosphorylation of IKK*β*, I*κ*B, and NF-*κ*B in A*β*-Activated BV2 Microglia

**Figure 4:**
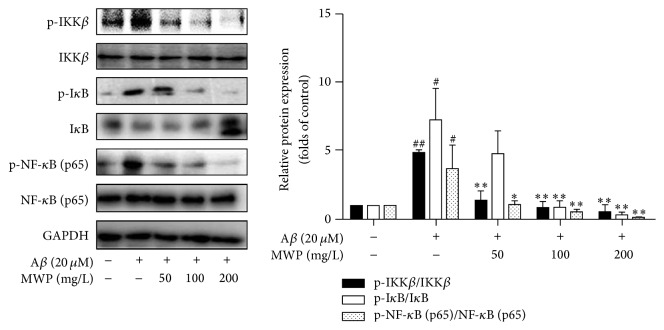
*MWP inhibits the NF-κB signaling pathway in Aβ-activated BV2 microglia*. BV2 microglia were incubated with or without MWP (50, 100, and 200 mg/L) for 30 min and stimulated with A*β* (20 *μ*M) for another 30 min. The total protein of the cells was then extracted and analyzed by western blot. Values are expressed as mean ± standard deviation of triplicate tests. ^#^*P* < 0.05 and ^##^*P* < 0.01 relative to the control group; ^*∗*^*P* < 0.05 and ^*∗∗*^*P* < 0.01 relative to the A*β* group.

After confirming that MWP inhibits NF-*κ*B (p65) nuclear translocation in A*β*-activated BV2 microglia, we investigated the effects of MWP on the activation of NF-*κ*B. As shown in Figure 4, A*β* (20 *μ*M) treatment markedly induced NF-*κ*B phosphorylation, which was significantly reversed by MWP. Next, we studied two crucial upstream proteins of the NF-*κ*B signaling pathway: I*κ*B kinase (IKK) and nuclear factor-*κ*B (I*κ*B). As shown in Figure 4, IKK*β* and I*κ*B were phosphorylated after A*β* treatment. MWP downregulated IKK*β* phosphorylation and inhibited I*κ*B degradation in a concentration-dependent manner. These results indicated that the NF-*κ*B signaling pathway is a potential therapeutic target in activated BV2 microglia.

### 3.5. MWP Inhibits ERK/p38 MAPK Signaling in A*β*-Activated BV2 Microglia

**Figure 5:**
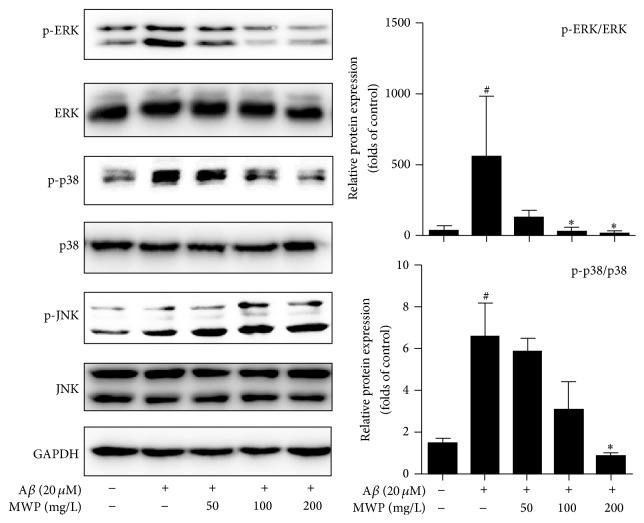
*MWP inhibits the ERK/p38 MAPK signaling pathway in Aβ-activated BV2 microglia*. BV2 microglia were incubated with or without MWP (50, 100, and 200 mg/L) for 30 min and stimulated with A*β* (20 *μ*M) for another 30 min. The total protein of the cells was extracted and analyzed by western blot. The values are expressed as mean ± standard deviation of triplicate tests. ^#^*P* < 0.05 relative to the control group; ^*∗*^*P* < 0.05 relative to the A*β* group.

We investigated the effects of MWP on the mitogen-activated protein kinase (MAPK) signaling pathway to determine whether MWP suppresses the inflammatory response in BV2 microglia via this pathway. As shown in Figure 5, A*β* (20 *μ*M) treatment increased the phosphorylation of extracellular regulated protein kinase (ERK) and p38 in BV2 microglia, which was significantly inhibited by MWP in a concentration-dependent manner. However, similar results were not observed with another MAPK family member, c-Jun NH2-terminal kinase (JNK). Thus, MWP could inhibit ERK/p38 MAPK signaling pathway in activated BV2 microglia.

## 4. Discussion

To date, there have been relatively few studies on the effects of traditional Chinese medicine on neuroinflammation. However, the extensive usage of Chinese herbs [17, 22] has made such studies necessary. Our team has been committed to the research of the anti-inflammatory function of the compound extracted from traditional Chinese medicine and the effect has been verified [23]. The result could also been considered the positive exploration of the pharmacological activity of traditional Chinese medicine. MWP is a traditional Chinese medicine prescription. In our previous work, we showed that MWP could protect rat astrocytes from damage due to neuroinflammation induced by LPS [22]. In this study, we aimed to determine whether MWP could inhibit neuroinflammation in A*β*-activated BV2 microglia and the mechanisms of its action.

AD is associated with upregulation of proinflammatory cytokines, which can initiate plaque production and enhance nerve cell degeneration [24]. Inflammatory factors are implicated in dementia-related peripheral disease and cerebral nervous system diseases [25]. Cerebral A*β* accumulation is the primary influence in AD [26]. Overactivated microglia could stimulate the immune system, which is overwhelmed by A*β* deposition. Inflammation then becomes chronic and adverse, resulting in accelerated neurodegeneration [7, 10]. In our study, the stimulation by A*β* (20 *μ*M) could increase the mRNA expression of IL-1*β*, IL-6, TNF-*α*, and MCP-1, which are common inflammatory factors. However, MWP treatment could inhibit this mRNA expression in a concentration-dependent manner in BV2 microglia. Moreover, treatment with MWP resulted in concentration-dependent attenuation of iNOS expression in BV2 microglia. All these results confirmed the potential of MWP in preventing neuroinflammation.

The NF-*κ*B signaling pathway is canonical in regulating inflammation. The activation of this pathway starts with the phosphorylation of upstream IKK. The protein I*κ*B, which is connected with NF-*κ*B, then degrades and separates. Activated NF-*κ*B exists as p-NF-*κ*B and is translocated to the nucleus to regulate the expression of inflammatory factors. As an important transcription factor, NF-*κ*B participates in the regulation of the immune system and inflammatory response [27]. NF-*κ*B binds to DNA, and its transcriptional activity regulates several genes, which promote neuroinflammation [28]. Microglia-mediated inflammation has been linked to the pathogenesis of CNS diseases [29]. Certain Chinese herbs or their extracts suppress the activation of BV2 microglia by inhibiting NF-*κ*B signaling [28, 30]. In our research, we found that A*β* could phosphorylate IKK*β*, I*κ*B, and NF-*κ*B and induce degradation of I*κ*B and nuclear translocation of NF-*κ*B. MWP evidently inhibited the expression of p-IKK*β*, p-I*κ*B, and p-NF-*κ*B in a concentration-dependent manner. The nuclear translocation of NF-*κ*B was also prevented. Thus, MWP could inhibit neuroinflammation by suppressing the activation of NF-*κ*B in A*β*-activated BV2 microglia.

MAPK is another signaling pathway that has been implicated in the mechanism of inflammation. ERK, p38, and JNK are among the most studied members of the MAPK family. Injection of A*β* in rat models could induce cognitive impairment and neuroinflammation by promoting nuclear translocation of NF-*κ*B and activating the ERK/p38 MAPK signaling pathway [31]. Additionally, microglia activation and proinflammatory responses occur by the modulation of a series of intracellular signaling pathways including IKK/I*κ*B and MAPK with the activation of A*β* [32, 33]. The prescription of herbs could improve A*β*-activated cognitive deficits through modulation of neuroinflammation by inhibiting ERK, JNK, and p38 MAPK pathways [34]. Thus, studies on the MAPK signaling pathway could provide a basis for the discovery of therapeutic targets to inhibit neuroinflammation. In our study, ERK, p38, and JNK were activated and phosphorylated by A*β*, while MWP inhibited the expression of p-ERK and p38. However, we did not achieve the same result with p-JNK. Therefore, MWP could inhibit the activation of ERK/p38 MAPK in A*β*-activated BV2 microglia.

Although quantities of data have proved the possible mechanisms of MWP in regulating the occurrence of AD, it is still a pity that the relevant clinical trials are rarely processed. It would be of great significance to conduct the population-based researches for the further exploration of the traditional application of MWP in remitting the generation and development of AD.

## 5. Conclusion

**Figure 6:**
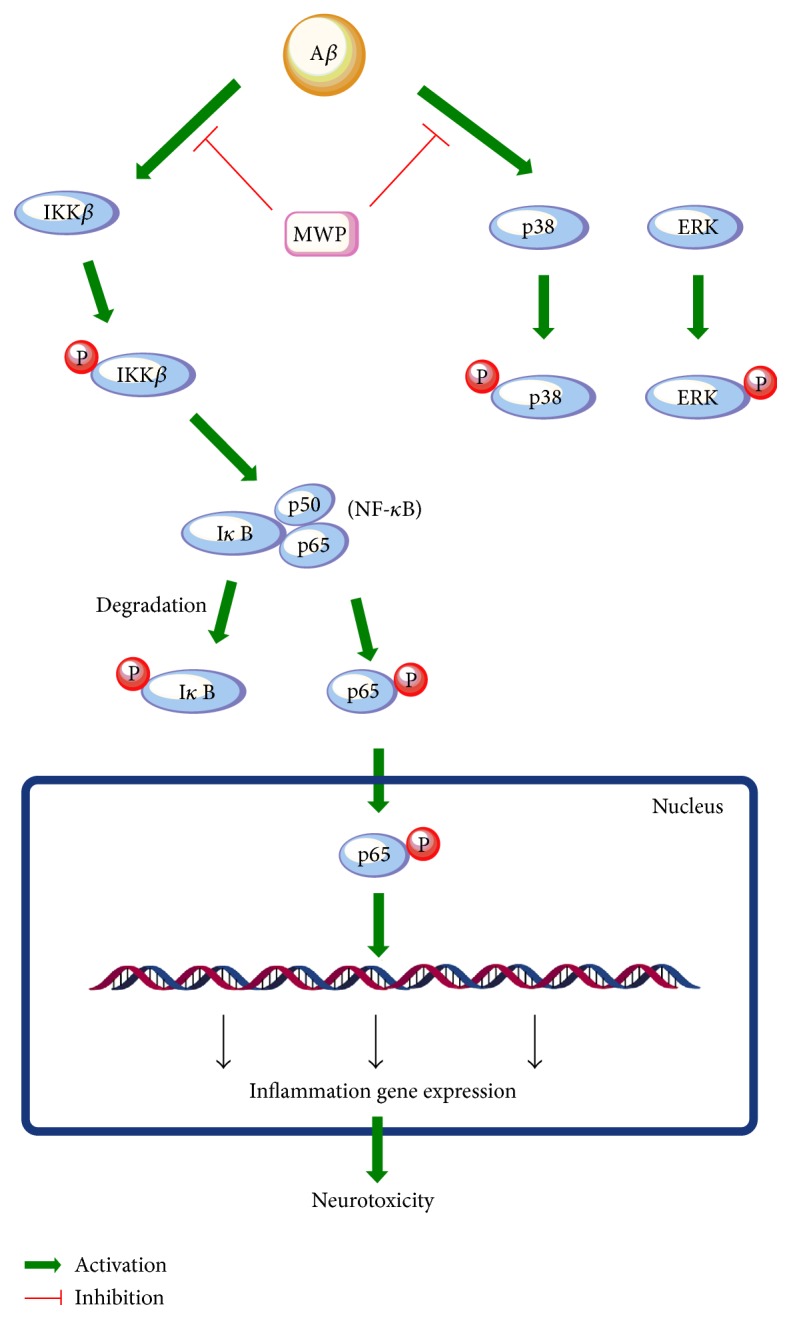
*Potential antineuroinflammatory mechanisms of MWP activity on activated BV2 microglia*. A*β* activates BV2 microglia via the IKK*β*/I*κ*B/NF-*κ*B and ERK/p38 MAPK signaling pathways, resulting in the activation of multiple proinflammatory mediators and the translocation of NF-*κ*B (p65) into the nucleus. MWP can inhibit the activation of these signaling pathways, thereby effecting antineuroinflammatory activity in BV2 microglia.

The traditional Chinese decoction MWP could effectively inhibit neuroinflammatory responses and the release of inflammatory factors such as IL-1*β*, IL-6, TNF-*α*, and MCP-1 and the expression of iNOS. This function was mainly obtained by the suppression of the NF-*κ*B and ERK/p38 MAPK signaling pathways (Figure 6). All our data indicated that MWP is a potential candidate in the treatment of neuroinflammation and merits further study.

## Figures and Tables

**Table 1:** Composition of modified Wu-Zi-Yan-Zong prescription (MWP).

Herbal composition	Part used	Amounts used (g)
*Lycium barbarum *L.	Fruit	400
*Cuscuta chinensis *Lam.	Fruit	400
*Rubus chingii *Hu.	Fruit	200
*Schisandra chinensis *(Turcz.) Baill.	Fruit	50
*Plantago asiatica *L.	Fruit	100
*Epimedium brevicornum *Maxim.	Herb	400

**Table 2:** *HPLC analysis of modified Wu-Zi-Yan-Zong prescription (MWP)*. Characterization of compounds in MWP by HPLC analysis. ^*∗*^Eb: *Epimedium brevicornum* Maxim.; Rc: *Rubus chingii* Hu; PA: *Plantago asiatica* L.; CC: *Cuscuta chinensis* Lam.; LB: *Lycium barbarum* L.; SC: *Schisandra chinensis* (Turcz.) Baill.

Number	Rt (min)	Identification	Constituent herbs^*∗*^					
			Eb	Rc	PA	SC	CC	LB
1	4.5	L-Glycero-*O*-galactoheptitol-3,6-anhydro-*O*-glucoside			+			
2	5.3	2-*α*-Nigerosylglucose						+
3	7.4	L-Ascorbic acid-*O*-glucoside			+			
4	18.5	Guanosine			+			+
5	38.9	Quinic acid-*O*-caffeoyl-*O*-glucoside			+			
6	41.5	Chlorogenic acid			+			
7	44.5	Quinic acid-*O*-caffeoyl-*O*-glucoside			+			
8	44.9	Limocitrol-*O*-glucoside	+					
9	46.2	Cinnamoylquinic acid			+			
10	51.2	Caffeoylquinic acid			+			
11	51.5	Caffeoylquinic acid			+			
12	55.5	Benzenepropanamide,N-[3-[[4-[(3-aminopropyl)amino] butyl]amino]^−^propyl]-3,4-dihydroxy						+
13	58.2	Cinnamoylquinic acid			+			
14	61.9	Kaempferol-di-*O*-glucoside	+	+	+		+	+
15	62.4	Quinic acid-tri-*O*-glucoside			+			
16	67.8	Quercetin-*O*-glucosyl-*O*-xyloside	+	+	+		+	+
17	81.6	Desmethylicaritin-*O*-rhamnosyl-*O*-glucoside	+					
18	82.1	Epimedoside E	+					
19	85.4	Hydroxyisocupressoside B				+	+	+
20	87.2	Sagittasine C	+					
21	87.9	Icariine	+					
22	88.6	Epimedin B	+					
23	89.5	Icaritin-*O*-rhamonosyl-*O*-rutinoside	+					
24	90.9	Epimediside A	+					
25	92.9	8-Dihydroprenylquercetin-*O*-methyl-tri-*O*-acetyl-*O*-rutinoside	+					
26	93.5	8-Dihydroprenylquercetin-di-*O*-cinnamoyl-*O*-glucoside	+					
27	99.2	Epimedin K	+					
28	100.2	Icaritin-tri-*O*-methyl-*O*-rutinoside	+					

**Table 3:** *MWP inhibits the mRNA expression of inflammatory mediators, as well as the protein expression of iNOS in Aβ-activated BV2 microglia*. The PCR primer of mRNA is shown in the table.

TNF-*α*	5′-3′ (forward) AGTGACAAGCCTGTAGCCCACGT
	5′-3′ (reverse) CCATCGGCTGGCACCACTAGTT
MCP-1	5′-3′ (forward) CTTCTGGGCCTGCTGTTCACAGTT
	5′-3′ (reverse) TTCTTGGGGTCAGCACAGACCTCT
IL-1*β*	5′-3′ (forward) TGGAGAAGCTGTGGCAGCTACCT
	5′-3′ (reverse) GAACGTCACACACCAGCAGGTT
IL-6	5′-3′ (forward) ACAAAGCCAGAGTCCTTCAGAGAGA
	5′-3′ (reverse) TGGTCTTGGTCCTTAGCCACTCCT
GAPDH	5′-3′ (forward) GGTGAAGGTCGGTGTGAACG
	5′-3′ (reverse) CTCGCTCCTGGAAGATGGTG
